# The Impact of COVID-19 Pandemic on Psychiatric Rehabilitation in Residential Facilities: Perspectives of Staff and Residents

**DOI:** 10.1007/s40737-023-00343-6

**Published:** 2023-04-24

**Authors:** Antonio Lasalvia, Luca Bodini, Camilla D’Astore, Francesca Gomez, Alessia Pesarin, Giuseppe Imperadore, Chiara Bonetto

**Affiliations:** 1 UOC Psichiatria, Azienda Ospedaliera Universitaria Integrata (AOUI) Di Verona, Policlinico “G.B. Rossi”, P.le Scuro, 10, 37134 Verona, Italy; 2 Section of Psychiatry, Department of Neuroscience, Biomedicine and Movement Sciences, University of Verona, Verona, Italy; 3 Department of Mental Health, ULSS 9 Scaligera, Verona, Italy

**Keywords:** Psychiatric residential facilities, Coronavirus, Healthcare workers, Mental disorder, Serious mental illness

## Abstract

The coronavirus pandemic imposed significant changes in the care of patients in psychiatric residential facilities, especially during lockdown periods. The aim of this study was to assess the impact of the pandemic on patients and staff of psychiatric residential facilities (RFs). This cross-sectional survey was conducted between 30 June and 30 July 2021 and involved 31 RFs located in the province of Verona (Italy). In total, 170 staff members and 272 residents participated in this study. Staff showing clinically significant symptoms of anxiety, depression and burnout were, respectively, 7.7%, 14.2% and 6%. Staff were concerned about the risk that COVID-19 infection might spread among residents (67.6%) and that residents could not receive an acceptable service due to service reconfiguration because of the pandemic (50.3%). Residents found it very unpleasant not to be permitted to attend family members (85.3%), and not to be allowed to engage in outdoor activities due to the restrictions (84%). Both staff and residents agree that the main problematic areas for the residents was the interdiction to meet family members or friends and the interdiction to outdoor activities, whereas problems related to the COVID-19 infection were considered by the staff members as more frequently problematic than reported by residents. The COVID-19 pandemic had a significant impact on the rehabilitation care and recovery journeys of the residents of psychiatric RFs. Therefore, sustained, and careful attention is needed to ensure that the rehabilitation needs of people with severe mental disorders are not neglected in time of pandemics.

**Supplementary Information:**

The online version contains supplementary material available at 10.1007/s40737-023-00343-6.

## Introduction

Italy was the first nation among the Western countries to be affected by the COVID-19 outbreak. Due to the rapid spread of the pandemic within the country, on 8 March 2020, the Italian government established stringent containment measures in Lombardy, Veneto, and some neighbouring provinces of Emilia-Romagna. On 11 March 2020, the Italian government imposed a nationwide lockdown. These extraordinary containment measures restricted the movement of everyone across the country, except for work or health reasons or in an exceptional case of necessity.

Starting from mid-March 2020, activities within hospitals in the most affected regions (e.g., Lombardy, Veneto, and Emilia Romagna) underwent a rapid and profound reorganisation to preserve beds and staff for COVID-19 patients (Faccincani et al., 2020; Marcon et al., 2020)*.* As a result, many hospital wards had to reorganise their activity, thus becoming COVID-19 wards. The COVID-19 pandemic posed an extraordinary strain on healthcare professionals working in secondary and tertiary hospitals and, more specifically, on those working in close contact with COVID-19 patients. Limited resources, longer shifts, disruptions to sleep and work–life balance, and occupational hazards associated with exposure to patients with COVID-19 have contributed to adverse psychological outcomes among healthcare workers in terms of burnout, post-traumatic stress, insomnia, anxiety, and depression (Lasalvia et al., 2020, 2021; Fagiolini et al., 2020; de Filippis et al, 2022).

In this context, mental health care also underwent significant changes: most psychiatric inpatient units—particularly in Lombardy, Emilia Romagna and Veneto—were closed and a significant proportion of psychiatrists, mental health nurses, and other healthcare workers were transferred to new COVID-19 wards (Carpiniello et al., 2020). During the lockdown period, local health authorities in most Italian regions, following national regulations, prescribed a reduction of ordinary outpatient and community mental health care to prevent the spread of the COVID-19 infection—only mental health care for most urgent cases was ensured (Percudani et al., 2020). Thus, most patients with chronic mental disorders who might need regular mental health care monitoring were deprived of these services. In addition, starting from mid-March 2020, day-care facilities for psychiatric patients were temporarily closed (in most regions they were only reopened but with a lot of restrictions in July 2020), whereas patients receiving residential mental health care were confined within facilities with no possibility to follow outdoor rehabilitative interventions (Martinelli et al., 2020). These measures significantly disrupted the vocational and psychosocial rehabilitation pathways of these patients. The philosophy of mental health rehabilitation focuses on helping persons deal with their social skills deficits through social skill training, encouraging social interactions and reducing social distance (Chaturvedi, 2020). Forced confinement within facilities might have negatively impacted on these patients’ clinical outcome, with the risk of aggravation of symptoms and even relapses. Moreover, because psychiatric residential facilities (RFs) were at high risk of infection (similarly to other long-term residential care facilities for care-dependent elderly people (de Girolamo et al, 2020) or children with intellectual disabilities (Xiong et al., 2020), staff had to quickly implement a series of actions and to adopt a range of containment measures to prevent the spread of the virus without no previous experience and training (i.e., in using personal protective equipment). This might have posed a particularly high strain on them and impacted on their psychological well-being.

The evaluation of the impact of the COVID-19 pandemic on psychiatric RFs has been substantially neglected by research at the international level. As far as we are aware, the only report on the impact of the pandemic on psychiatric RFs was carried out within the broader context of a survey of the perspectives and experiences of staff working in inpatient and community settings across the UK health and social care sectors (Johnson et al., 2021). However, this survey did not assess the impact of the pandemic as perceived by the residents of residential facility themselves. A study that was conducted on a small group of patients receiving care within a psychiatric residential facility near Rome (Italy) only focused on their mental health status, without providing any information on the changes that occurred in their rehabilitation pathways due to the pandemic (Cordellieri et al., 2021). This paper aims to fill this gap by analysing the impact of the COVID-19 pandemic on psychiatric RFs located in the province of Verona (Italy), according to the perceptions of both staff and residents.

### Psychiatric RFs in Italy and in Veneto Region

Psychiatric RFs represent a crucial component of the Italian mental health care system. Healthcare in Italy is provided by the National Health System on a regional basis. Mental health care is also provided on a regional basis, with a model that is community-based across the national territory. In each region, the Mental Health Department (MHD) is responsible for psychiatric care to the adult population living in a well-defined geographical area and has a range of services and facilities, including community mental health centres, day-care facilities, general hospital psychiatric units and RFs. These non-hospital community facilities are functionally linked to the MHD serving the same geographical area and generally host patients from the same area or neighbouring areas. Overall, psychiatric RFs provide residential rehabilitation interventions aiming to help residents developing practical living skills and to promote their personal recovery, independence, and social inclusion. Rehabilitative programs are expected to be tailored to individual needs, personalized and periodically updated, and should address not only practical daily care and nursing but also engage patients in meaningful daily activities and societal participation (Martinelli et al., 2020). Patients receiving care within psychiatric RFs have generally severe and complex mental health problems (such as schizophrenia and other psychoses), with associated cognitive difficulties that impair their ability to manage activities of daily living (Martinelli et al, 2019). According to the Italian Mental Health Report, at the time of the survey addressed in this paper Italian psychiatric RFs hosted an overall number of 27.800 residents (Ministero della Salute, 2022).

Psychiatric RFs in Italy may be broadly classified into three main types, according to the intensity of care and staffing level. In the Veneto region the most intensive type of RF is the Comunità Terapeutica Riabilitativa Protetta (sheltered therapeutic rehabilitation facility; CTRP), which is a psychiatric facility that is designed to meet healthcare needs of younger patients requiring short or medium term rehabilitative residential care. They are generally located in large buildings with 8 to 14 beds and staff are available onsite 24 h a day. The second type of RF is the Comunità Alloggio (community sheltered houses); these RFs are classified as Comunità Alloggio Estensiva (extensive community sheltered houses; CAE) and Comunità Alloggio di Base (basic community sheltered houses; CAB) and aim to provide residential rehabilitation care for older patients with higher and lower needs, respectively. More specifically, CABs are designed to support for autonomy and self-management of more independent and autonomous residents; they usually have 4–6 places and staff are available for a maximum of 12 h a day; CAEs provide intermediate-high intensity assistance for residents with more chronic, severe, and stable mental disorder; they usually serve 12–20 residents and staff are available 24 h a day (Martinelli et al., 2022). The third kind of RFs are called Gruppo Appartamento Protetto (GAP), which are basically home groups that simulate the typical family life. These RFs consist of small apartments that are located in the community and are tailored for the most autonomous patients (generally 3 or 4 for each apartment), who are encouraged to take an active role in the maintenance of the household, such as performing chores or helping to manage a budget. Staff are usually available for no more than 4 h a day.

## Method

### Study Design

A cross-sectional survey was conducted between 30 June and 30 July 2021 within psychiatric RFs located in the province of Verona (Veneto region, northeast Italy), an area of approximately 925,000 inhabitants. Data collection on staff working in RFs was conducted using a web-based questionnaire hosted on the Lime survey platform. The online survey required about 15 to 20 min to be completed. The study description, invitation to participate, and a link to the online questionnaire were sent via e-mail to all staff. Three reminders for completing the questionnaire were sent after the first, the second and the third week. The survey was anonymous, and confidentiality of information was granted. All participants provided informed consent.

Residents receiving care within each participating RF were approached by a member of the research staff who explained the purpose of the study, gave full details in writing, and made it clear that participation was voluntary—the participants were told that they could choose whether to participate or not, or to participate and withdraw later. The residents were included in the study only after informed written consent had been gained. Those consenting to participate responded anonymously to the questionnaire that, once completed, was consigned to the facility coordinator in a closed envelope that was sent by mail to the research staff. Therefore, confidentiality was fully preserved. The residents’ questionnaires required about 10 to 15 min to be completed. The survey was approved by the Ethics Committee of the Provinces of Verona and Rovigo (approval No. 31656, 25th May 2021).

### Study Samples

At the time of the investigation, 44 psychiatric RFs were operating in the province of Verona. Specifically, 8 were CTRP, 9 were CAE, 6 were CAB, and 21 were GAP. Overall, 70.5% RFs (n = 31) participated in this study (8 CTRP, 8 CAE, 6 CAB, and 9 GAP). As compared to the total number RFs, GAPs were underrepresented in this survey. Regarding the staff, the eligible population was composed of 310 workers, of whom 170 (54.8%) participated in this survey. As regards residents, 407 were receiving rehabilitation care within psychiatric RFs in the province of Verona. Specifically, at the time of investigation, 109 residents (26.8%) were receiving treatment within CTRP, 150 (36.9%) in CAE, 55 (13.5%) in CAB, and 93 (22.8%) in GAP. Overall, 272 (66.8%) residents consented to participate. Of these, 78 (20.7%) were in CTRP, 104 (38.2%) in CAE, 44 (16.9%) in CAB, and 46 (16.2%) in GAP. Percentages of residents stratified by type of RF substantially overlap between participating sample and the eligible population.

### Assessment Measures

#### Information Collected Among Staff

A set of standardised measures was used to assess the mental health of staff working within RFs participating in the study.

Anxiety was assessed by the General Anxiety Disorder scale (GAD-7) (Spitzer et al., 2006), a 7-item self-rated questionnaire where each item is rated on a four-point scale, ranging from 0 (not at all) to 3 (nearly every day). In this study we adopted a cut-off score of 10 that represents a reasonable cut point for identifying those showing at least moderate symptoms of anxiety.

Depression was assessed by the Patient Health Questionnaire (PHQ-9) (Kroenke et al., 2001), a self-rated 9-item scale that asks if the subject has experienced symptoms of depression in the previous two weeks. Subjects are asked to rate how often each symptom occurred, ranging from 0 (not at all) to 3 (nearly every day). We used a cut-off score of 10 to indicate a condition potentially deserving clinical attention (Kroenke et al., 2001).

Burnout was assessed by the Maslach Burnout Inventory-General Survey (MBI-GS) (Schaufeli & Van Dierendonck, 1993), a modified version of the original MBI that was designed to be used in a wide range of occupational settings. MBI-GS consists of 16 items constituting three subscales: Emotional Exhaustion (EX), Cynicism (CY) and Professional Efficacy (EF). All MBI-GS items are scored on a 7-point rating scale ranging from 0 (never) to 6 (always). The cut-off scores for the three MBI-GS subscales, tested on large sample of Italian healthcare professionals (Lasalvia et al., 2021), were, respectively, > 2.20 for EX, > 2.00 for CY and < 3.66 for EF. Burnout was defined as having a high EX and, at the same time, a high CY or a low EF.

Two ad hoc questionnaires were used to collect information on challenges found at work by staff of RFs during the COVID-19 pandemic (19 items) and problems faced by residents of RFs from a staff perspective (8 items). Both questionnaires (whose responses were rated on a 5-point Likert scale ranging from 1 “not relevant” to 5 “extremely relevant”) were developed by the research staff based on the instruments produced by the COVID-19 Mental Health Policy Research Unit Group (Johnson et al., 2021).

Personal socio-demographic information and job-related characteristics were also collected, including gender, age, living condition, having psychological problems developed before the COVID-19 outbreak requiring specialised help, occupation, length of working experience, and place of work.

#### Information Collected Among Residents

Information on the residents’ perspective on the changes that occurred within RFs during the COVID-19 pandemic was collected by using an ad hoc schedule that was developed by the research group together with four members of a users’ association based in south Verona—*Il Cerchio Aperto* (The Open Circle). Item generation was performed within structured focus groups sessions, which were run by experienced clinicians and researchers. This is a self-reported schedule that is composed of 17 items, exploring the residents’ perceptions of a range of circumstances of daily life within RFs that might have been affected by the pandemic. Responses are rated on a three-point scale (1-unpleasant, 2-neutral, 3-pleasant).

### Statistical Analysis

Categorical variables were described by frequencies and percentages; for continuous variables, means (standard deviations) and ranges were given. The association between categorical characteristics was checked by using Chi-square test (or Fisher’s exact test in the case of 2 × 2 contingency tables), with a significant *p*-value of 0.05. For staff, percentages of those scoring above the cut-off score in each mental health outcome [GAD-7 ≥ 10; PHQ-9 ≥ 10, MBI-EX > 2.2, MBI-CY > 2.0, MBI-EF < 3.66 and MBI total (EX > 2.2, CY > 2.0 and EF < 3.66)] were stratified by personal (sex, age, living condition, marital status, education) and job characteristics [type of occupation, length of working experience (< 6 yrs., 6–20 yrs., > 20 yrs.), workplace (CAB, CAE, CTRP, GAP)]. For residents, percentages of those reporting unpleasant perceptions related to COVID-19 pandemic in the various daily life domains (i.e., scoring “1” in the ad hoc questionnaire) were stratified for sex, years since illness onset (≤ 10 yrs,11–20 yrs., 21–30, > 30 yrs.), length of stay in residential facility (≤ 5 yrs., 6–10 yrs.,11–15 yrs., > 15 yrs.) and typology of residential facility (CAB, CAE, CTRP, GAP). Analyses were performed by SPSS 28 for Windows.

## Results

### Characteristics of the Participating Staff

The personal and job-related characteristics of participating staff (n = 170) are given in Table 1.

**Table 1:** Personal and job characteristics of staff working in RFs (n = 170)

		n	%
Sex (1 missing)	Female	120	71.0
Age	< 36 yrs	35	20.6
	36–55 yrs	96	56.5
	> 55 yrs	39	22.9
Living condition	With partner and children	66	38.8
	With partner	44	25.9
	Alone	26	15.3
	With other relatives	18	10.6
	With children but no partner	16	9.4
Marital status (2 missing)	Married or cohabiting	109	64.9
	Single or non-cohabiting partner	37	22.0
	Widowed, separated or divorced	22	13.1
Education	Primary or secondary school	31	18.2
	Diploma	78	45.9
	Degree or postgraduate qualification	61	35.9
Occupation	Healthcare assistant	103	60.6
	Support worker	24	14.1
	Other healthcare staff	20	11.8
	Nurse	15	8.8
	Psychiatric rehabilitation therapist	8	4.7
Length of working experience	< 6 yrs	46	27.1
	6–20 yrs	71	41.7
	> 20 yrs	53	31.2
Workplace*	GAP	23	13.5
	CAB	21	12.4
	CAE	67	39.4
	CTRP	59	34.7

*GAP home groups; CAB basic community sheltered houses; CAE extensive community sheltered houses; CTRP sheltered therapeutic rehabilitation facility

In brief, nearly three quarters of the respondents were females (71%), half were aged 36 to 55 years (56.5%), most were married or cohabiting (64.9%) and lived with other people (84.7%). Most staff described themselves as health care assistants (60.6%), 16.5% as unqualified healthcare staff, 14.1% as psychiatric rehabilitation therapists, and 8.8% as psychiatric nurses. Overall, the participants were experienced staff because most (72.9%) had been working in the mental health sector for at least 6 years. Most staff worked in CAEs and CTRPs (74.1%).

### Mental Health Outcomes of Participating Staff

Overall, 7.7% of participating staff had developed clinically significant symptoms of anxiety (GAD-7 ≥ 10; 15 missing) and 14.2% symptoms of at least moderate depression (PHQ-9 ≥ 10; 15 missing). Regarding MBI-GS, high scores on emotional exhaustion (EX > 2.20; 19 missing) and cynicism (CY > 2.00; 19 missing) were reported, respectively, by 12.6% and 11.3%, while low scores on professional efficacy (EF < 3.66; 19 missing) by 31.1%; only 6% (n = 19 missing) displayed a condition of burnout. No significant differences were found across the mental health outcomes considered by stratifying for personal, job-related characteristics and place of work (i.e., typology of residential facility) (see the on-line Supplementary Part 1).

### Staff Perception of Changes Related to the COVID-19 Pandemic

Table 2 shows challenges faced at work by participating staff due to the pandemic.

**Table 2:** Challenges faced by staff of RFs during the COVID-19 pandemic (n = 170)

	Extremely relevant	Very relevant	Moderately relevant	Slightly relevant	Not relevant
	n (%)	n (%)	n (%)	n (%)	n (%)
*Organisational and job-related issues*					
Increased difficulty managing work-life balance (missing 16)	14 (19.5)	30 (21.4)	44 (28.6)	33 (21.4)	33 (21.4)
Increased workload due to staff shortages (missing 17)	24 (15.7)	30 (19.6)	37 (24.2)	40 (26.1)	22 (14.4)
Having to adapt too quickly to new ways of working (missing 16)	21 (13.6)	42 (27.3)	50 (32.5)	27 (17.5)	14 (9.1)
Pressures resulting from the need to support colleagues through the stresses associated with the pandemic (missing 15)	18 (11.6)	36 (23.2)	50 (32.3)	28 (18.1)	23 (14.8)
Working in a new condition with different organisational, emotional and care problems from usual (missing 15)	16 (10.3)	54 (34.8)	50 (32.3)	28 (18.1)	7 (4.5)
Having to learn to use new technologies too quickly and/or without sufficient training and support (missing 16)	7 (4.5)	25 (16.2)	37 (24.0)	47 (30.5)	38 (24.7)
Pressure to accept redeployment to a setting where I don't feel happy to work (missing 19)	2 (1.3)	4 (2.6)	8 (5.3)	19 (12.6)	118 (78.1)
*Control of infection*					
The risk that COVID-19 would spread among residents (missing 16)	36 (23.4)	68 (44.2)	35 (22.7)	7 (4.5)	8 (5.2)
Difficulty maintaining infection control because residents did not understand or were too unwell to follow procedures (missing 15)	21 (13.5)	38 (24.5)	56 (36.1)	26 (16.8)	14 (9.0)
Difficulty maintaining infection control because residents could not be effectively segregated from one another in this environment (missing 15)	18 (11.6)	47 (30.3)	53 (34.2)	27 (17.4)	10 (6.5)
Difficulty managing communal areas of accommodation safely (missing 15)	14 (9.0)	46 (29.7)	55 (35.5)	25 (16.1)	15 (9.7)
Challenges supporting residents who were very worried about COVID-19 infection (missing 16)	6 (3.9)	32 (20.8)	62 (40.3)	36 (23.4)	18 (11.7)
Difficulty getting appropriate medical care for residents who are ill with COVID-19 infections (missing 21)	2 (1.3)	14 (9.4)	30 (20.1)	29 (19.5)	74 (49.7)
*Consequences on residents*					
Residents not getting an acceptable service due to service reconfiguration because of COVID-19 (missing 15)	20 (12.9)	58 (37.4)	49 (31.6)	20 (12.9)	8 (5.2)
More challenging environment because residents could not go out and engage in outdoor activities as usual (missing 15)	18 (11.6)	53 (34.2)	61 (39.4)	20 (12.9)	3 (1.9)
Not being able to have as much contact as usual with residents due to staff shortages or changes in service offered (missing 15)	9 (5.8)	33 (21.3)	51 (32.9)	34 (21.9)	28 (18.1)
*Relationship with other services*					
Lack of support because of closure of or reduction in community mental health services (missing 18)	15 (9.9)	48 (31.6)	38 (25.0)	29 (19.1)	22 (14.5)
Lack of support due to reduction in other services in the community e.g., primary care. social care. voluntary sector services (missing 17)	12 (7.8)	53 (34.6)	43 (28.1)	28 (18.3)	17 (11.1)
Lack of support and expertise by healthcare services in managing physical health problems in patients infected with Covid-19 (missing 22)	6 (4.1)	21 (14.2)	34 (23.0)	29 (19.6)	58 (39.2)

Most participants (67.6%) were extremely or very concerned about the risk that COVID-19 infection might spread among residents, whereas half of them (50.3%) were extremely or very concerned that residents could not receive an acceptable service due to service reconfiguration because of the COVID-19 pandemic. Other extremely or very relevant issues reported by participants were related to the more challenging environment within the facilities because the residents could not go out and engage in outdoor activities (45.8%); working in a new condition with unusual organisational, emotional and care problems (45.1%); lack of support due to reduction in other services in the community (42.4%); difficulty in maintaining infection control as residents could not be segregated from one another (41.9%); lack of support because of closure or reduction in community mental health services (41.5%); increased difficulty managing work-life balance (40.9%); and having to adapt too quickly to new ways of working (40.9%).

Table 3 shows the staff perceptions of the main problems faced by residents due to the pandemic.

**Table 3:** Staff perspectives of the problems faced by the residents (n = 170)

	Extremely relevant	Very relevant	Moderately relevant	Slightly relevant	Not relevant
	N (%)	N (%)	N (%)	N (%)	N (%)
Lack of access to usual support networks of family and friends (16 missing)	42 (27.3)	69 (44.8)	21 (13.6)	16 (10.4)	6 (3.9)
Worries about getting COVID-19 infection (16 missing)	26 (16.9)	47 (30.5)	54 (35.1)	17 (11.0)	10 (6.5)
Lack of usual work and outdoor activities (17 missing)	21 (13.7)	62 (40.5)	41 (26.8)	21 (13.7)	8 (5.2)
Relapse and/or deterioration in mental health triggered by the COVID-19 stress (16 missing)	11 (7.1)	38 (24.7)	52 (33.8)	29 (18.8)	24 (15.6)
High personal risk of severe consequences of COVID-19 infection (e.g., due to physical health comorbidities) (18 missing)	9 (5.9)	29 (19.1)	32 (21.1)	39 (25.7)	43 (28.3)
Effects of COVID-19-related trauma (17 missing)	8 (5.2)	27 (17.6)	51 (33.3)	37 (24.2)	30 (19.6)
Problems with neighbours because of lack of understanding of/ability to stick to government requirements (16 missing)	2 (1.3)	11 (7.1)	12 (7.8)	39 (25.3)	90 (58.4)
Problems with police or other authorities because of lack of understanding of/ability to stick to government requirements (16 missing)	0 (0.0)	1 (0.6)	18 (11.7)	31 (20.1)	104 (67.5)

Most of the participating staff (72.1%) agreed that the lack of access to usual support networks of family and friends might represent an extremely or very relevant problem for residents. Furthermore, more than half of participants (54.2%) reported that lack of usual work and outdoor activities due to the COVID-19 restrictions represented an extremely or very relevant problem for residents. Nearly half of the participants (47.4%) believed that worries about getting COVID-19 infection was an extremely or very relevant problem for residents. Nearly 32% of staff were also extremely or highly concerned that COVID-related stress might trigger relapse and/or deterioration in the residents’ mental health.

### Characteristics of the Participating Residents

Table 4 reports the socio-demographic and clinical information of the participating residents (n = 272).

**Table 4:** Socio-demographic and clinical characteristics of residents participating in the study (n = 272)

Sex, n (%)	
Male	168 (61.8)
Age, n (%)	
≤ 40 yrs	54 (19.9)
41–50 yrs	53 (19.5)
51–60 yrs	116 (42.6)
> 60 yrs	49 (18.0)
Self-reported clinical diagnosis, n (%)	
Schizophrenia	111 (40.8)
Bipolar disorder	35 (12.9)
Personality disorder	30 (11.1)
Non-schizophrenic psychosis	47 (17.3)
Schizoaffective disorder	21 (7.7)
Delusional disorder	10 (3.7)
Major depression	8 (2.9)
Obsessive compulsive disorder	5 (1.8)
Other	5 (1.8)
Length of stay in RF (yrs.), mean (SD); min–max	5.8 (5.2); 0–23
Years since illness onset, mean (SD); min–max	20.2 (11.1); 1–51

Most participating residents were male (61.8%), aged over 41 years (80.1%). Their average length of stay within RFs was nearly 6 years, whereas average illness duration was 20 years. The most represented diagnostic groups, as reported by the residents themselves, were schizophrenia and related disorders (40.8%), bipolar disorders (12.9%) and personality disorders (11.1%).

### Residents’ Perceptions of the Challenges Related to the COVID-19 Pandemic

Table 5 shows the residents’ perceptions of the main problems that they faced within RFs due to the pandemic.

**Table 5:** Residents’ perspectives of the changes that occurred within psychiatric RFs during the COVID-19 pandemic (*“How did you consider….?”*) (n = 272)

	Unpleasant	Neutral	Pleasant
	n (%)	n (%)	n (%)
*Relationship with family and/or friends*			
Not being allowed to join family gatherings/family celebrations (e.g., holidays, anniversary, birthday parties) or outdoor activities organised by friends and/or family (NA 12, missing 1)	221 (85.3)	24 (9.3)	14 (5.4)
Not being allowed to meet friends and/or family members (NA 4)	225 (84.0)	29 (10.8)	14 (5.2)
*Outdoor activities*			
To spend all the time within the facility (or in your room) due to interdiction of outdoor activities (NA 6)	216 (81.2)	34 (12.8)	16 (6.0)
Not being allowed to go shopping to practice and improve social skills trained during rehabilitation activities provided within the facility (NA 54, missing 5)	173 (81.2)	28 (13.1)	12 (5.6)
Not being allowed to engage in outdoor activities (walking/hiking. volunteering. sports. cycling trips. day trip/excursions) (NA 6, missing 1)	214 (80.8)	37 (14.0)	14 (5.3)
Not being allowed to attend work, education or vocational training programmes due to interdiction of outdoor activities (NA 183, missing 6)	65 (78.3)	13 (15.7)	5 (6.0)
Not being allowed to use public transport to practice and improve social skills trained during rehabilitation activities provided within the facility (NA 157, missing 5)	83 (75.5)	19 (17.3)	8 (7.3)
Not being able to attend usual planned activities at day centres due to closure of facilities (NA 179, missing 8)	50 (58.8)	19 (22.4)	16 (18.8)
*Indoor activities*			
Not being allowed to have leisure activities together with other residents within the facility (e.g., playing games, watching movies/TV, listening to music, eating together, having a party) due to physical distancing measures (NA 33)	168 (70.3)	52 (21.8)	19 (7.9)
Not being allowed to do something helpful for other residents due to physical distancing measures (NA 104, missing 3)	107 (64.8)	40 (24.2)	18 (10.9)
To have been requested to adopt physical distancing of at least 1 m from others while participating to rehabilitation activities within the facility (NA 17, missing 2)	151 (59.7)	76 (30.0)	26 (10.3)
*Organisational changes*			
The overall impact of the COVID-19 pandemic and related restrictive measures on the quality of your life (NA 9, missing 3)	202 (77.7)	34 (13.1)	24 (9.2)
To engage with remote consultations by phone or via digital platforms with GPs, treating psychiatrists or other therapists (NA 130, missing 2)	82 (58.6)	28 (20.0)	30 (21.4)
To be exclusively engaged in indoor rehabilitation activities due to interdiction of outdoor activities (NA 13, missing 3)	149 (58.2)	54 (21.1)	53 (20.7)
*Control of infection*			
To adopt preventive measures within the facility, such as wear facial mask. use hand sanitiser gel, undergo triage procedures (NA 4, missing 2)	158 (59.4)	76 (28.6)	32 (12.0)
To regularly undergo nasopharyngeal swab (NA 12, missing 3)	120 (46.7)	70 (27.2)	67 (26.1)
To have the chance of being first in line for COVID-19 vaccination as a resident of a healthcare facility (NA 10, missing 2)	43 (16.5)	42 (16.2)	175 (67.3)

The great majority of the residents found that it was very unpleasant that they were not allowed to join family gatherings, family celebrations or outdoor activities organised by friends and/or family members (85.3%), and that they were not allowed to meet their friends and/or family members (84%) due to the pandemic restrictions. Moreover, most residents found that it was very unpleasant to spend all the time locked in the facility (81.2%), to not be allowed to go shopping to practice social skills learned in therapy sessions (81.2%), to not be allowed to engage in outdoor activities to generalise social skills in community settings (78.3%), to not be allowed to use public transport to practice social skills learned in therapy sessions (75.5%). Overall, nearly 80% reported that restrictive measures adopted during the pandemic had significant negative impact on their quality of life.

By stratifying the percentages of dissatisfaction in the various areas by personal and clinical characteristics of residents or by type of residential facility, some significant associations emerged. Specifically, dissatisfaction was more frequent in the questions evaluating: (a) interdiction to go shopping for females, for those with less than 10 or between 21 and 30 years since illness onset and for those living in CTRPs; (b) interdiction to have leisure activities together with other residents within facility for those with 11–20 years since illness onset and for those living in CABs; (c) interdiction to engage in outdoor activities for females and for those with 11–20 years since illness onset; (d) interdiction to attend work, education or vocational training for those with length of stay of 6–10 years in RFs and for those living in CABs; (e) interdiction to use public transport for females; (f) interdiction to attend activities at day centres for those with less than 10 years since illness onset; (g) engagement with remote consultations with GPs, psychiatrists, or other therapists for those with length of stay of 6–15 years in RFs; (h) obligation to regularly undergo nasopharyngeal swabs for residents of CABs (Fisher’s exact or Chi-square tests where appropriate, *p* < 0.05) (see the on-line Supplementary Part 2 for details).

### Comparison Between the Perceptions of the Staff and Residents

**Fig. 1:**
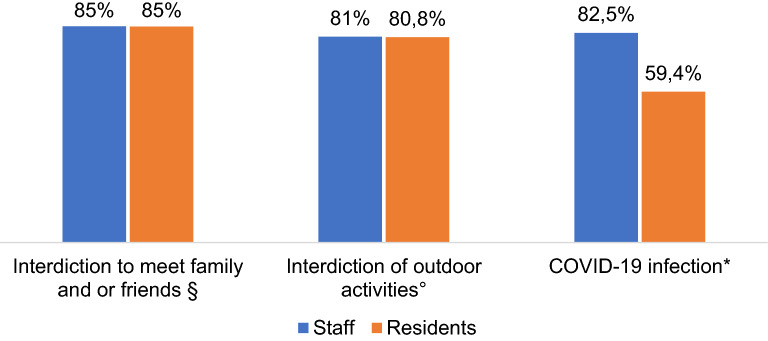
Comparison of staff’s perspectives of the problems of the residents and the problems reported by residents on the most three challenging areas (percentages of subjects reporting challenges/problems in the three considered areas are given). § For staff, the item considered was “Lack of access to usual support networks of family and friends” (responses "Extremely relevant”, “Very relevant” and “Moderately relevant” were summed). For residents, the item considered was “How did you consider not being allowed to join family gatherings/ family celebrations or outdoor activities organised by friends and /or family?” (response “Unpleasant”). *°* For staff the item considered was “Lack of usual work and outdoor activities” (responses "Extremely relevant”. “Very relevant” and “Moderately relevant” were summed). For residents the item considered was “How did you consider not being allowed to engage in outdoor activities?” (response “Unpleasant”). * For staff the item considered was “Worries about getting COVID-19 infection” (responses "Extremely relevant”. “Very relevant” and “Moderately relevant” were summed). For residents the item considered was “How did you consider adopting preventive measures within the facility, such as wear facial mask, use hand sanitiser gel, undergo triage procedures?” (response “Unpleasant”)

Figure 1 shows a comparison of the staff perception of main problems faced by residents and the problems reported by residents themselves on the three main comparable domains.

Both staff and residents agree that the main problematic areas for residents was the interdiction to meet family members or friends (nearly 85%) and the interdiction to outdoor activities (nearly 81%), whereas problems related to the COVID-19 infection were considered by the staff members to be more frequently problematic (82.5%) than reported by residents (59.4%) (comparison on this latter domain it should be taken with caution as the corresponding items of the two questionnaires are not exactly the same).

## Discussion

This study helps to shed some light on the impact of the COVID-19 pandemic on psychiatric RFs, as perceived by staff and residents. To the best of our knowledge, this is the first study to address this issue. Most research on the impact of the pandemic on healthcare workers was conducted on hospital workers (more frequently those at frontline with COVID-19) (Sanghera et al., 2020) or other health care professionals working in the community, such as general practitioners (Jefferson et al., 2022) or staff working within nursing homes for elderly people (Palacios-Ceña et al., 2021) or long-term residential facilities for people with intellectual disabilities (Chen et al., 2022). Unfortunately, the mental health of healthcare professionals working within psychiatric RFs has been neglected. Similarly, the impact of the COVID-19 pandemic on patients receiving residential mental health rehabilitation—who represent one of the most vulnerable segments of the population—has been largely neglected by research.

We found that the COVID-19 pandemic did not significantly impact on mental health of staff working within psychiatric RFs. Only a small fraction of staff in our study reported symptoms of clinically meaningful depression, anxiety, and burnout. The percentages of staff reporting clinically significant symptoms of general anxiety, depression and burnout in this study were remarkably lower than those found among healthcare staff working in a tertiary hospital located in the same geographical area during the pandemic (Lasalvia et al., 2020) and among a sample of general practitioners (Lasalvia et al., 2022) working in the same area. This is not an unexpected finding because healthcare workers, particularly those at the frontline with COVID-19 patients, experienced a wide range of stressful and/or definitely traumatic events at work (e.g., undergoing sudden reassignment to other hospital units or new unfamiliar tasks, working under increased workload conditions, having to deal with a great number of deaths in a relatively short time, seeing patients dying alone as relatives were not allowed to enter the restricted areas or communicating by telephone the death of a beloved one to relatives, etc.) that might significantly impact on their mental health (Sanghera et al., 2020). It is noteworthy that the percentage of RF staff showing clinically significant symptoms of anxiety and depression was far lower than that found in the general population in Italy (Amerio et al., 2021), which suggests that these professionals display good resilience skills. Alternatively, given that this study was conducted in a period when the number of new COVID-19 cases were relatively low and the Italian epidemic curve had flattened, we may hypothesise that our findings are more conservative and optimistic than those collected during the lockdown or post-lockdown periods.

The COVID-19 pandemic was challenging in many ways for staff working in psychiatric RFs, who had to face several organisational changes and adapt to them quickly. We found that one major concern reported by staff was to implement all the required containment measures within their facilities to prevent or reduce infections among the residents. This was a problematic issue indeed because most of the staff working in psychiatric RFs were unqualified healthcare workers who had no previous experience or sufficient training on how to use personal protective equipment or to apply and follow appropriate infection control practices and procedures. Moreover, to prevent contagion within the facilities, the staff had to minimise personal contact with the residents and had to ensure that the residents would respect social distancing measures (thus avoiding any personal interaction), which had a negative impact on the perception of quality of care provided. In fact, most staff expressed concerns that residents would not receive an acceptable service due to service reconfiguration during to the COVID-19 pandemic. A further burden on staff working within psychiatric RFs was the lack of support by other services in the community (e.g., primary care, social care, voluntary sector) and/or by community mental health services. Unfortunately, most community-based mental health services in northern Italy were requested to stay closed or to reduce their activity during the COVID-19 pandemic, with a drastic reduction of home visits or visits to patients within RFs (Carpiniello & Vita, 2022). Increased difficulty in managing work-life balance was another theme that was expressed as a major concern by staff. This is a relevant issue because the many of the workers reported that it was difficult to balance work and leisure time, which represents a main factor for developing job-related distress and burnout (Pattnaik et al., 2022). Organisations are responsible for providing a conducive, positive and healthy work environment for their employees, especially in a time of crisis.

We found that the COVID-19 pandemic was particularly burdensome for residents of psychiatric RFs. The main problem for most residents was the interpersonal isolation that they were requested by the restrictive measures adopted to prevent the spread of infection. This implied the prohibition of visits by friends or relatives, or to join family meetings and outdoor gatherings with friends. The prohibition of outdoor activities (e.g., going to work, to school, training sessions or other occupational activities) was the other main problem reported by most residents. Another major problem reported by residents was the obligation to practice interpersonal distancing within the facilities. The residents reported that what they most missed during the COVID-19 restrictions period were group therapies, informal social activities with other residents and other forms of peer-interactions that together represent a central component of the personal recovery process (Slade et al., 2014). Thus, for residents of RFs, the challenges they have faced in the COVID-19 pandemic mirror those reported for the general population in terms of a sense of isolation and of being locked down in their facilities (Rossi et al., 2020). They also reported specific impacts on their rehabilitation care and recovery journeys. Group interactions and outdoor projects are an inherent part of their treatment, such as when they walk the grounds, dine in communal areas, watch television together in day rooms, exercise and go to therapy together. Peer-support groups meetings (i.e., groups of residents who gather to share and discuss common problems and experiences, led by a professional or volunteer discussion leader or facilitator) and informal activities are a vital source of emotional and spiritual support to people who struggle to stay in personal recovery (Slade et al, 2014). Hence, isolation can be very dangerous for these patients and the fear of contracting a life-threatening illness is unlikely to promote personal recovery (Aamir et al., 2021). Having to stay within the facility would not only slow down the progress in social skills development but would also reduce their self-reliance and self-confidence and affect their vocational potential.

It is interestingly that some gender differences emerged when analysing the various daily life domains within facilities that were most impacted by the pandemic, as female residents expressed more dissatisfaction with the impossibility to generalise the social skills learnt in therapy sessions to other settings (e.g., to go shopping, to attend work, education or vocational training, to use public transport) due to the interdiction of outdoor activities. This finding seems to provide further support to the importance of implementing gender-sensitive recovery-oriented interventions within rehabilitation services (Mizock, 2019; Dixon et al., 2022).

The staff responses had very similar themes to the residents’ responses when asked what in their view might have impacted most on the residents. In fact, the staff was aware that a lack of access to the usual support networks of family and friends might have represented an extremely relevant problem for residents. Furthermore, staff shared with residents the worry about the negative impact of the restrictions on specific rehabilitation interventions, particularly the disruption of usual work, training and outdoor activities. This is a positive finding and is an indirect indicator of a good therapeutic relationship between staff and residents.

### Limitations

This study has several limitations. First, it was not possible to establish whether both staff and resident samples were representative of the respective populations (this specifically applies to staff, as detailed information on characteristics of the eligible population was not available). Thus, caution should be exercised when generalising our results. Second, the results from this study cannot be generalised to other mental health rehabilitation services (i.e., day-care services) or to the broader population of people with severe mental illness because the sample addressed here was recruited within residential rehabilitation services. Third, mental health status was assessed on staff members only, whereas no formal assessment of mental health status was performed on residents because we were only interested to evaluate the impact of the pandemic on their daily life and rehabilitation pathways. Fourth, organisational information on participating RFs was self-reported and may possess declaration biases. Fifth, the participants completed the survey retrospectively, which may have introduced the risk of recall bias. Finally, the ad hoc questionnaire used to collect information on the residents’ perspective on the changes occurring within RFs during the COVID-19 pandemic did not undergo formal validation.

### Clinical Implications and Future Directions

Once the containment measures prescribed the closure of outdoor activities and the interpersonal distancing, the different types of RFs lost any specificity. In fact, the most problematic issue posed by the pandemic as perceived by both the staff and the residents was the burden of being locked away from family, friends and loved ones, without any possibility to meet anyone else except their treating staff. This was common across the different typologies of RFs. None of the other problematic issues due to the pandemic differed across the typology of RFs. Indeed, the different typology of RFs in Veneto are supposed to have substantial specificities in terms of intensity of rehabilitative care and staffing level (with CTRPs having the highest, while GAPs the lowest) and in terms of type of interventions provided (mainly healthcare interventions within CTRPs, mainly forms of social support in GAPs). It thus seems that the pandemic, with its closures and containment measures, somehow homogenised the different typologies of the RFs. This represents a sort of natural experiment that reminds us of the sense and meaning of psychiatric residential rehabilitation—without any projection toward the outside, toward the community they are in, psychiatric RFs are likely to lose any specificity and any real rehabilitative potential (de Girolamo et al., 2005). Psychiatric RFs, which in Italy are conceived and designed as non-hospital community facilities where residents are free to come and go during the day, must be open to the outside world to promote full social integration and personal recovery. Otherwise, they would only be useless closed boxes, some sort of seclusion facility or a new form of institutionalisation.

On the other hand, it should be underlined that in times of healthcare crisis, such as the COVID-19 pandemic, any effort to avoid the spread of infection by reducing social contacts and by closing facilities to the outside world might also have had a positive effect as it probably saved lives of many residents. Further studies are needed to help policymakers and administrators to balance the pros and cons of closing this kind of facilities, taking into consideration the therapeutic and psychological consequences for residents.

## Conclusion

The COVID-19 pandemic had a significant impact on rehabilitation care and recovery journeys of residents of psychiatric RFs. This is a particularly relevant issue. The substantial decrease in psychosocial and rehabilitative interventions for such a prolonged time is not likely to be without consequences for the mental health status of this population. However, the detrimental effect of disruption of rehabilitative interventions will probably manifest in a later stage, in the long run. Therefore, sustained and careful attention is needed to ensure that the rehabilitation needs of people with severe mental disorders are not neglected in the focus on maintaining the health and well-being of the population and of other vulnerable groups in time of pandemics.

## Supplementary Information

Below is the link to the electronic supplementary material.

Supplementary file1 (DOCX 19 KB)

Supplementary file2 (DOCX 37 KB)

## References

[CR1] Aamir A, Awan S, de Filippis R, Diwan MN, Ullah I (2021). Effect of COVID-19 on mental health rehabilitation centers. Journal of Psychosocial Rehabilitation and Mental Health.

[CR2] Amerio A, Lugo A, Stival C, Fanucchi T, Gorini G, Pacifici R, Odone A, Serafini G, Gallus S (2021). COVID-19 lockdown impact on mental health in a large representative sample of Italian adults. Journal of Affective Disorders.

[CR3] Carpiniello, B., & Vita, A. (2022). Impact of COVID-19 on the Italian Mental Health System: A Narrative Review. *Schizophrenia Bulletin Open*, 3(1), sgac038. 10.1093/schizbullopen/sgac038 PMC9619790 36348642

[CR4] Carpiniello B, Tusconi M, Zanalda E, Di Sciascio G, Di Giannantonio M (2020). Psychiatry during the Covid-19 pandemic: A survey on mental health departments in Italy. BMC Psychiatry.

[CR5] Chaturvedi SK (2020). Covid-19, coronavirus and mental health rehabilitation at times of crisis. Journal of Psychosocial Rehabilitation and Mental Health.

[CR6] Chen, Y., Allen, A. P., Fallon, M., Mulryan, N., McCallion, P., McCarron, M., & Sheerin, F. (2022). The challenges of mental health of staff working with people with intellectual disabilities during COVID-19––A systematic review. *Journal of Intellectual Disabilities*, 17446295221136232. 10.1177/17446295221136231 PMC9606641 36285537

[CR7] Cordellieri P, Barchielli B, Masci V, Viani F, de Pinto I, Priori A, Torriccelli FD, Cosmo C, Ferracuti S, Giannini AM (2021). Psychological health status of psychiatric patients living in treatment communities before and during the COVID-19 lockdown: A brief report. International Journal of Environmental Research and Public Health.

[CR8] de Girolamo, G., Picardi, A., Santone, G., Falloon, I., Morosini, P., Fioritti, A., Micciolo, R. & PROGRES Group. (2005). The severely mentally ill in residential facilities: a national survey in Italy. *Psychological Medicine* 35(3), 421–431 10.1017/s0033291704003502 15841877

[CR9] de Filippis R, Soler-Vidal J, Pereira-Sanchez V, Ojeahere MI, Morimoto K, Chang A, Schuh Teixeira A.L., Spadini AV (2022). Coronavirus outbreak from early career psychiatrists' viewpoint: What we have learned so far. Perspectives in Psychiatric Care.

[CR10] de Girolamo G, Bellelli G, Bianchetti A, Starace F, Zanetti O, Zarbo C, Micciolo R (2020). Older people living in long-term care facilities and mortality rates during the COVID-19 pandemic in Italy: Preliminary epidemiological data and lessons to learn. Frontiers in Psychiatry.

[CR11] Dixon K, Fossey E, Petrakis M (2022). Using photovoice to explore women's experiences of a women-only prevention and recovery care service in Australia. Health and Social Care Community.

[CR12] Faccincani R, Pascucci F, Lennquist S (2020). How to surge to face the SARS-CoV-2 outbreak: Lessons learned from Lombardy, Italy. Disaster Medicine and Public Health Preparedness.

[CR13] Fagiolini, A., Cuomo, A., & Frank, E. (2020). COVID-19 diary from a psychiatry department in Italy. *Journal of Clinical Psychiatry,* 81(3):20com13357. 10.4088/JCP.20com13357 32237301

[CR14] Jefferson L, Golder S, Heathcote C, Avila AC, Dale V, Essex H, van der Feltz Cornelis C, McHugh E, Moe-Byrne T, Bloor K (2022). GP wellbeing during the COVID-19 pandemic: A systematic review. British Journal of General Practice.

[CR15] Johnson, S., Dalton-Locke, C., Vera San Juan, N., Foye, U., Oram, S., Papamichail, A., Landau, S., Rowan Olive, R., Jeynes, T., & Shah, P. (2021). Impact on mental health care and on mental health service users of the COVID-19 pandemic: A mixed methods survey of UK mental health care staff.*Social Psychiatry and Psychiatric Epidemiology*, 56(1), 25–37. 10.1007/s00127-020-01927-4 PMC7453694 32857218

[CR16] Kroenke K, Spitzer RL, Williams JB (2001). The PHQ-9: Validity of a brief depression severity measure. Journal of General Internal Medicine.

[CR17] Lasalvia A, Bonetto C, Porru S, Carta A, Tardivo S, Bovo C, Ruggeri M, Amaddeo F (2020). Psychological impact of COVID-19 pandemic on healthcare workers in a highly burdened area of north-east Italy. Epidemiology and Psychiatric Sciences.

[CR18] Lasalvia A, Amaddeo F, Porru S, Carta A, Tardivo S, Bovo C, Ruggeri M, Bonetto C (2021). Levels of burn-out among healthcare workers during the COVID-19 pandemic and their associated factors: A cross-sectional study in a tertiary hospital of a highly burdened area of north-east Italy. BMJ Open.

[CR19] Lasalvia A, Rigon G, Rugiu C, Negri C, Del Zotti F, Amaddeo F, Bonetto C (2022). The psychological impact of COVID-19 among primary care physicians in the province of Verona, Italy: A cross-sectional study during the first pandemic wave. Family Practice.

[CR20] Marcon G, Tettamanti M, Capacci G, Fontanel G, Spanò M, Nobili A, Forloni G, Franceschi C (2020). COVID-19 mortality in Lombardy: The vulnerability of the oldest old and the resilience of male centenarians. Aging.

[CR21] Martinelli A, Iozzino L, Pozzan T, Cristofalo D, Bonetto C, Ruggeri M (2022). Performance and effectiveness of step progressive care pathways within mental health supported accommodation services in Italy. S Social Psychiatry and Psychiatric Epidemiology.

[CR22] Martinelli A, Iozzino L, Ruggeri M, Marston L, Killaspy H (2019). Mental health supported accommodation services in England and in Italy: A comparison. Social Psychiatry and Psychiatric Epidemiology.

[CR23] Martinelli A, Ruggeri M (2020). The impact of COVID-19 on patients of Italian mental health supported accommodation services. Social Psychiatry and Psychiatric Epidemiology.

[CR24] Ministero della Salute. (2022). *Rapporto salute mentale. Analisi dei dati del Sistema Informativo per la Salute Mentale (SISM). Anno 2021*.

[CR25] Mizock L (2019). Development of a gender-sensitive and recovery-oriented intervention for women with serious mental illness. PSychiatric Rehabilitation Journal.

[CR26] Palacios-Ceña D, Fernández-Peña R, Ortega-López A, Fernández-Feito A, Bautista-Villaécija O, Rodrigo-Pedrosa O, Arnau-Sánchez J, Lizcano-Álvarez Á (2021). Long-term care facilities and nursing homes during the first wave of the COVID-19 pandemic: A scoping review of the perspectives of professionals, families and residents. International Journal of Environmental Research and Public Health.

[CR27] Pattnaik, T., Samanta, S., & Mohanty, J. (2022). Work Life Balance of Health Care Workers in the New Normal: A Review of Literature. *Journal of Medicinal and Chemical Sciences*, 42–54.

[CR28] Percudani M, Corradin M, Moreno M, Indelicato A, Vita A (2020). Mental health services in Lombardy during COVID-19 outbreak. Psychiatry Research.

[CR29] Rossi, R., Socci, V., Talevi, D., Mensi, S., Niolu, C., Pacitti, F., Di Marco, A., Rossi, A., Siracusano, A., & Di Lorenzo, G. (2020). COVID-19 pandemic and lockdown measures impact on mental health among the general population in Italy. *Frontiers in Psychiatry*, 790. 10.3389/fpsyt.2020.00790 PMC7426501 32848952

[CR30] Sanghera J, Pattani N, Hashmi Y, Varley KF, Cheruvu MS, Bradley A, Burke JR (2020). The impact of SARS-CoV-2 on the mental health of healthcare workers in a hospital setting-A systematic review. Journal of Occupational Health.

[CR31] Schaufeli WB, Van Dierendonck D (1993). The construct validity of two burnout measures. Journal of Organizational Behavior.

[CR32] Slade M, Amering M, Farkas M, Hamilton B, O'Hagan M, Panther G, Perkins R, Shepherd G, Tse S, Whitley R (2014). Uses and abuses of recovery: implementing recovery-oriented practices in mental health systems. World Psychiatry.

[CR33] Spitzer RL, Kroenke K, Williams JBW, Löwe B (2006). A brief measure for assessing generalized anxiety disorder: The GAD-7. Archives of Internal Medicine.

[CR34] Xiong GL, Atkin A, Moquin K, Candido M, Beilenson P, Kasirye O, Wasserman M, Blum P, Hilty D (2020). COVID-19 transmission in a psychiatric long-term care rehabilitation facility: An observational study. Primary Care Companion for CNS Disorders.

