# Supplementary material for: The Impact of COVID-19 Pandemic on Psychiatric Rehabilitation in Residential Facilities: Perspectives of Staff and Residents
Source: J Psychosoc Rehabil Ment Health. 2023 Apr 24:1–15. Online ahead of print. doi: 10.1007/s40737-023-00343-6 (PMC10123581; doi:10.1007/s40737-023-00343-6)
Supplement: Supplementary file 1 — Supplementary file1 (DOCX 19 KB) [file 40737_2023_343_MOESM1_ESM.docx]

**Online Supplementary Part 1**

**Table 1.** Association between each mental health outcome and personal characteristics for participating staff (n=170)

|  | **Staff characteristics** | | | | |
| --- | --- | --- | --- | --- | --- |
|  | **Sex** | **Age** | **Living condition*** | **Marital status^#^** | **Education^§^** |
| GAD-7 ≥10 | M 6.4%; F 8.4% | <36 3.1%; 36-55 7.0%; >55 13.5% | A 4.0%; P 9.8%; PC 10.2%; C 0.0%; OR 5.9% | S 5.9%; MC 10.2%; WSD 0.0% | PS 0.0%; D 11.3%; DP 7.3% |
| PHQ ≥10 | M 10.6%; F 15.0% | <36 12.5%; 36-55 14.0%; >55 16.2% | A 12.0%; P 14.6%; PC 18.6%; C 0.0%; OR 11.8% | S 8.8%; MC 17.3%; WSD 9.1% | PS 13.8%; D 19.7%; DP 7.3% |
| MBI EX >2.20 | M 8.5%; F 14.6% | <36 9.4%; 36-55 9.6%; >55 22.2% | A 12.5%; P 20.0%; PC 13.8%; C 0.0%; OR 0.0% | S 0.0%; MC 16.7%; WSD 14.3% | PS 13.8%; D 13.2%; DP 11.1% |
| MBI CY >2.00 | M 17.0%; F 8.7% | <36 9.4%; 36-55 12.0%; >55 11.1% | A 16.7%; P 10.0%; PC 8.6%; C 15.4%; OR 12.5% | S 12.1%; MC 9.4%; WSD 19.0% | PS 6.9%; D 11.8%; DP 13.0% |
| MBI EF <3.66 | M 38.3%; F 28.2% | <36 28.1%; 36-55 31.3%; >55 33.3% | A 29.2%; P 40.0%; PC 29.3%; C 30.8%; OR 18.8% | S 30.3%; MC 33.3%; WSD 19.0% | PS 44.8%; D 22.1%; DP 35.2% |
| MBI Burnout | M 6.4%; F 5.8% | <36 3.1%; 36-55 4.8%; >55 11.1% | A 4.2%; P 10.0%; PC 6.9%; C 0.0%; OR 0.0% | S 0.0%; MC 8.3%; WSD 4.8% | PS 10.3%; D 5.9%; DP 3.7% |

* PC With partner and children, P With partner, A Alone, OR With other relatives, C With children but no partner

# S Single or non-cohabiting partner, MC Married or cohabiting, WSD Widowed, separated or divorced

§ PS Primary or secondary school, D Diploma, DP Degree or postgraduate qualification

p-value (Chi-square or Fisher’s exact test; due to low frequencies for a number of cells, the tests were executed for explorative purposes)

Sex: GAD-7 1.00, PHQ 0.613, MBI EX 0.429, MBI CY 0.167, MBI EF 0.256, MBI Burnout 1.000

Age: GAD-7 0.253, PHQ 0.913, MBI EX 0.136, MBI CY 0.920, MBI EF 0.897, MBI Burnout 0.308

Living condition: GAD-7 0.666, PHQ 0.509, MBI EX 0.181, MBI CY 0.841, MBI EF 0.601, MBI Burnout 0.520

Marital status: GAD-7 0.244, PHQ 0.356, MBI EX 0.063, MBI CY 0.443, MBI EF 0.437, MBI Burnout 0.213

Education: GAD-7 0.158, PHQ 0.139, MBI EX 0.918, MBI CY 0.695, MBI EF 0.062, MBI Burnout 0.476

**Table 2.** Association between each mental health outcome and job-related characteristics for participating staff (n=170)

|  | **Staff characteristics** | | |
| --- | --- | --- | --- |
|  | **Occupation*** | **Length of working experience** | **Workplace** |
| GAD-7 ≥10 | OH 5.9%; N 13.3%; S 8.7%; H 7.5%; P 0.0% | <6 7.0%; 6-20 6.5%; >20 10.0% | CAB 5.9%; CAE 11.1%; CTRP 3.6%; GAP 10.0% |
| PHQ ≥10 | OH 11.8%; N 20.0%; S 4.3%; H 17.2%; P 0.0% | <6 18.6%; 6-20 16.1%; >20 8.0% | CAB 11.8%; CAE 15.9%; CTRP 14.5%; GAP 10.0% |
| MBI EX >2.20 | OH 0.0%; N 21.4%; S 17.4%; H 13.2%; P 0.0% | <6 12.2%; 6-20 11.7%; >20 14.0% | CAB 5.9%; CAE 15.9%; CTRP 7.8%; GAP 20.0% |
| MBI CY >2.00 | OH 0.0%; N 28.6%; S 8.7%; H 11.0%; P 14.3% | <6 9.8%; 6-20 10.0%; >20 14.0% | CAB 17.6%; CAE 14.3%; CTRP 5.9%; GAP 10.0% |
| MBI EF <3.66 | OH 37.5%; N 42.9%; S 34.8%; H 28.6%; P 14.3% | <6 31.7%; 6-20 30.0%; >20 32.0% | CAB 41.2%; CAE 27.0%; CTRP 33.3%; GAP 30.0% |
| MBI Burnout | OH 0.0%; N 21.4%; S 4.3%; H 5.5%; P 0.0% | <6 4.9%; 6-20 5.0%; >20 8.0% | CAB 5.9%; CAE 6.3%; CTRP 5.9%; GAP 5.0% |

* H Healthcare assistant, S Support worker, OH Other healthcare staff, N Nurse, P Psychiatric rehabilitation therapist

p-value (Chi-square or Fisher’s exact test; due to low frequencies for a number of cells, the tests were executed for explorative purposes)

Occupation: GAD-7 0.851, PHQ 0.382, MBI EX 0.306, MBI CY 0.168, MBI EF 0.636, MBI Burnout 0.108

Length of working experience: GAD-7 0.765, PHQ 0.293, MBI EX 0.931, MBI CY 0.754, MBI EF 0.971, MBI Burnout 0.757

Workplace: GAD-7 0.471, PHQ 0.914, MBI EX 0.340, MBI CY 0.427, MBI EF 0.698, MBI Burnout 0.997
