# Supplementary material for: The Impact of COVID-19 Pandemic on Psychiatric Rehabilitation in Residential Facilities: Perspectives of Staff and Residents
Source: J Psychosoc Rehabil Ment Health. 2023 Apr 24:1–15. Online ahead of print. doi: 10.1007/s40737-023-00343-6 (PMC10123581; doi:10.1007/s40737-023-00343-6)
Supplement: Supplementary file 2 — Supplementary file2 (DOCX 37 KB) [file 40737_2023_343_MOESM2_ESM.docx]

**Online Supplementary Part 2**

**Table 1.** Association between each challenge related to the COVID-19 pandemic and sex for residents (n=272)

|  | M  Unpleasant | F  Unpleasant | p-value  Fisher’s exact |
| --- | --- | --- | --- |
|  | % | % |  |
| **Relationship with family and/or friends** |  |  |  |
| Not being allowed to join family gatherings/family celebrations (e.g., holidays, anniversary, birthday parties) or outdoor activities organised by friends and/or family (NA 12, missing 1) | 82.8% | 89.6% | 0.150 |
| Not being allowed to meet friends and/or family members (NA 4) | 81.8% | 87.4% | 0.305 |
| **Outdoor activities** |  |  |  |
| To spend all the time within the facility (or in your room) due to interdiction of outdoor activities (NA 6) | 82.8% | 78.6% | 0.423 |
| Not being allowed to go shopping to practice and improve social skills trained during rehabilitation activities provided within the facility (NA 54, missing 5) | 76.5% | 88.9% | 0.030 |
| Not being allowed to engage in outdoor activities (walking/hiking. volunteering. sports. cycling trips. day trip/excursions) (NA 6, missing 1) | 76.1% | 88.2% | 0.016 |
| Not being allowed to attend work, education or vocational training programmes due to interdiction of outdoor activities (NA 183, missing 6) | 75.0% | 85.2% | 0.398 |
| Not being allowed to use public transport to practice and improve social skills trained during rehabilitation activities provided within the facility (NA 157, missing 5) | 68.9% | 88.9% | 0.032 |
| Not being able to attend usual planned activities at day centres due to closure of facilities (NA 179, missing 8) | 53.1% | 66.7% | 0.266 |
| **Indoor activities** |  |  |  |
| Not being allowed to have leisure activities together with other residents within the facility (e.g., playing games, watching movies/TV, listening to music, eating together, having a party) due to physical distancing measures (NA 33) | 69.8% | 71.1% | 0.884 |
| Not being allowed to do something helpful for other residents due to physical distancing measures (NA 104, missing 3) | 63.6% | 67.3% | 0.730 |
| To have been requested to adopt physical distancing of at least 1 metre from others while participating to rehabilitation activities within the facility (NA 17, missing 2) | 56.1% | 65.3% | 0.151 |
| **Organisational changes** |  |  |  |
| The overall impact of the COVID-19 pandemic and related restrictive measures on the quality of your life (NA 9, missing 3) | 78.6% | 76.2% | 0.650 |
| To engage with remote consultations by phone or via digital platforms with GPs, treating psychiatrists or other therapists (NA 130, missing 2) | 55.6% | 62.7% | 0.487 |
| To be exclusively engaged in indoor rehabilitation activities due to interdiction of outdoor activities (NA 13, missing 3) | 60.9% | 53.7% | 0.295 |
| **control of Infection** |  |  |  |
| To adopt preventive measures within the facility, such as wear facial mask. use hand sanitiser gel, undergo triage procedures (NA 4, missing 2) | 62.0% | 55.3% | 0.307 |
| To regularly undergo nasopharyngeal swab (NA 12, missing 3) | 47.8% | 45.0% | 0.702 |
| To have the chance of being first in line for COVID-19 vaccination as a resident of a healthcare facility (NA 10, missing 2) | 17.5% | 15.0% | 0.732 |

**Table 2.** Association between each challenge related to the COVID-19 pandemic and years since illness onset for residents (n=272)

|  | ≤10 yrs  Unpleasant | 11-20 yrs  Unpleasant | 21-30 yrs  Unpleasant | >30 yrs  Unpleasant | p-value  Chi-square |
| --- | --- | --- | --- | --- | --- |
|  | % | % | % | % |  |
| **Relationship with family and/or friends** |  |  |  |  |  |
| Not being allowed to join family gatherings/*f*amily celebrations (e.g., holidays, anniversary, birthday parties) or outdoor activities organised by friends and/or family (NA 12, missing 1) | 88.6% | 86.2% | 77.8% | 79.1% | 0.405 |
| Not being allowed to meet friends and/or family members (NA 4) | 84.0% | 85.0% | 81.5% | 79.1% | 0.866 |
| **Outdoor activities** |  |  |  |  |  |
| To spend all the time within the facility (or in your room) due to interdiction of outdoor activities (NA 6) | 79.6% | 75.9% | 83.6% | 74.4% | 0.669 |
| Not being allowed to go shopping to practice and improve social skills trained during rehabilitation activities provided within the facility (NA 54, missing 5) | 87.2% | 78.0% | 88.6% | 58.3% | 0.004 |
| Not being allowed to engage in outdoor activities (walking/hiking. volunteering. sports. cycling trips. day trip/excursions) (NA 6, missing 1) | 73.5% | 89.3% | 81.8% | 67.4% | 0.043 |
| Not being allowed to attend work, education or vocational training programmes due to interdiction of outdoor activities (NA 183, missing 6) | 73.3% | 80.8% | 81.0% | 71.4% | 0.898 |
| Not being allowed to use public transport to practice and improve social skills trained during rehabilitation activities provided within the facility (NA 157, missing 5) | 65.4% | 80.8% | 82.6% | 60.0% | 0.316 |
| Not being able to attend usual planned activities at day centres due to closure of facilities (NA 179, missing 8) | 80.0% | 68.2% | 52.9% | 18.2% | 0.006 |
| **Indoor activities** |  |  |  |  |  |
| Not being allowed to have leisure activities together with other residents within the facility (e.g., playing games, watching movies/TV, listening to music, eating together, having a party) due to physical distancing measures (NA 33) | 51.3% | 79.6% | 74.5% | 70.0% | 0.025 |
| Not being allowed to do something helpful for other residents due to physical distancing measures (NA 104, missing 3) | 51.7% | 67.5% | 68.8% | 53.8% | 0.372 |
| To have been requested to adopt physical distancing of at least 1 metre from others while participating to rehabilitation activities within the facility (NA 17, missing 2) | 42.6% | 61.8% | 64.7% | 65.9% | 0.075 |
| **Organisational changes** |  |  |  |  |  |
| The overall impact of the COVID-19 pandemic and related restrictive measures on the quality of your life (NA 9, missing 3) | 72.0% | 80.4% | 73.1% | 73.8% | 0.745 |
| To engage with remote consultations by phone or via digital platforms with GPs, treating psychiatrists or other therapists (NA 130, missing 2) | 55.2% | 54.3% | 56.0% | 69.6% | 0.660 |
| To be exclusively engaged in indoor rehabilitation activities due to interdiction of outdoor activities *(*NA 13, missing 3) | 64.4% | 50.8% | 56.0% | 52.4% | 0.543 |
| **control of Infection** |  |  |  |  |  |
| To adopt preventive measures within the facility, such as wear facial mask. use hand sanitiser gel, undergo triage procedures (NA 4, missing 2) | 47.1% | 62.7% | 71.7% | 55.8% | 0.070 |
| To regularly undergo nasopharyngeal swab (NA 12, missing 3) | 37.5% | 43.6% | 45.1% | 44.2% | 0.871 |
| To have the chance of being first in line for COVID-19 vaccination as a resident of a healthcare facility (NA 10, missing 2) | 8.0% | 13.8% | 21.6% | 16.7% | 0.282 |

**Table 3.** Association between each challenge related to the COVID-19 pandemic and length of stay in a residential facility for residents (n=272)

|  | ≤5 yrs  Unpleasant | 6-10 yrs  Unpleasant | 11-15 yrs  Unpleasant | >15 yrs  Unpleasant | p-value  Chi-square |
| --- | --- | --- | --- | --- | --- |
|  | % | % | % | % |  |
| **Relationship with family and/or friends** |  |  |  |  |  |
| Not being allowed to join family gatherings/*f*amily celebrations (e.g., holidays, anniversary, birthday parties) or outdoor activities organised by friends and/or family (NA 12, missing 1) | 81.8% | 92.5% | 87.5% | 85.0% | 0.223 |
| Not being allowed to meet friends and/or family members (NA 4) | 80.9% | 89.4% | 95.8% | 76.2% | 0.112 |
| **Outdoor activities** |  |  |  |  |  |
| To spend all the time within the facility (or in your room) due to interdiction of outdoor activities (NA 6) | 80.5% | 85.1% | 83.3% | 71.4% | 0.554 |
| Not being allowed to go shopping to practice and improve social skills trained during rehabilitation activities provided within the facility (NA 54, missing 5) | 80.0% | 90.7% | 63.2% | 80.0% | 0.060 |
| Not being allowed to engage in outdoor activities (walking/hiking. volunteering. sports. cycling trips. day trip/excursions) (NA 6, missing 1) | 77.9% | 84.8% | 91.7% | 76.2% | 0.305 |
| Not being allowed to attend work, education or vocational training programmes due to interdiction of outdoor activities (NA 183, missing 6) | 78.2% | 93.8% | 83.3% | 33.3% | 0.024 |
| Not being allowed to use public transport to practice and improve social skills trained during rehabilitation activities provided within the facility (NA 157, missing 5) | 73.0% | 87.5% | 57.1% | 80.0% | 0.327 |
| Not being able to attend usual planned activities at day centres due to closure of facilities (NA 179, missing 8) | 54.0% | 77.3% | 50.0% | 44.4% | 0.215 |
| **Indoor activities** |  |  |  |  |  |
| Not being allowed to have leisure activities together with other residents within the facility (e.g., playing games, watching movies/TV, listening to music, eating together, having a party) due to physical distancing measures (NA 33) | 66.7% | 71.9% | 80.0% | 80.0% | 0.435 |
| Not being allowed to do something helpful for other residents due to physical distancing measures (NA 104, missing 3) | 65.3% | 64.3% | 75.0% | 56.3% | 0.783 |
| To have been requested to adopt physical distancing of at least 1 metre from others while participating to rehabilitation activities within the facility (NA 17, missing 2) | 55.1% | 68.3% | 65.2% | 60.0% | 0.321 |
| **Organisational changes** |  |  |  |  |  |
| The overall impact of the COVID-19 pandemic and related restrictive measures on the quality of your life (NA 9, missing 3) | 78.3% | 71.9% | 87.5% | 80.0% | 0.445 |
| To engage with remote consultations by phone or via digital platforms with GPs, treating psychiatrists or other therapists (NA 130, missing 2) | 50.6% | 77.8% | 75.0% | 50.0% | 0.037 |
| To be exclusively engaged in indoor rehabilitation activities due to interdiction of outdoor activities (NA 13, missing 3) | 58.4% | 56.9% | 52.2% | 68.4% | 0.752 |
| **control of Infection** |  |  |  |  |  |
| To adopt preventive measures within the facility, such as wear facial mask. use hand sanitiser gel, undergo triage procedures (NA 4, missing 2) | 58.3% | 59.1% | 66.7% | 60.0% | 0.895 |
| To regularly undergo nasopharyngeal swab (NA 12, missing 3) | 47.0% | 45.3% | 52.2% | 42.9% | 0.929 |
| To have the chance of being first in line for COVID-19 vaccination as a resident of a healthcare facility (NA 10, missing 2) | 11.7% | 23.8% | 26.1% | 20.0% | 0.081 |

**Table 4.** Association between each challenge related to the COVID-19 pandemic and typology of residential facility for residents (n=272)

|  | CAB  Unpleasant | GAP  Unpleasant | CAE  Unpleasant | CTRP  Unpleasant | p-value  Chi-square |
| --- | --- | --- | --- | --- | --- |
|  | % | % | % | % |  |
| **Relationship with family and/or friends** |  |  |  |  |  |
| Not being allowed to join family gatherings/*f*amily celebrations (e.g., holidays, anniversary*,* birthday parties) or outdoor activities organised by friends and/or family (NA 12, missing 1) | 79.1% | 88.4% | 86.3% | 85.9% | 0.625 |
| Not being allowed to meet friends and/or family members (NA 4) | 88.6% | 80.4% | 82.2% | 85.7% | 0.670 |
| **Outdoor activities** |  |  |  |  |  |
| To spend all the time within the facility (or in your room) due to interdiction of outdoor activities (NA 6) | 81.4% | 83.7% | 79.8% | 81.6% | 0.956 |
| Not being allowed to go shopping to practice and improve social skills trained during rehabilitation activities provided within the facility (NA 54, missing 5) | 76.5% | 83.8% | 73.2% | 93.3% | 0.019 |
| Not being allowed to engage in outdoor activities (walking/hiking. volunteering. sports. cycling trips. day trip/excursions) (NA 6, missing 1) | 79.5% | 78.6% | 84.2% | 78.2% | 0.741 |
| Not being allowed to attend work, education or vocational training programmes due to interdiction of outdoor activities (NA 183, missing 6) | 95.5% | 81.8% | 56.3% | 73.9% | 0.032 |
| Not being allowed to use public transport to practice and improve social skills trained during rehabilitation activities provided within the facility (NA 157, missing 5) | 76.0% | 73.9% | 80.0% | 73.0% | 0.933 |
| Not being able to attend usual planned activities at day centres due to closure of facilities (NA 179, missing 8) | 58.3% | 57.1% | 58.8% | 60.7% | 0.995 |
| **Indoor activities** |  |  |  |  |  |
| Not being allowed to have leisure activities together with other residents within the facility (e.g., playing games, watching movies/TV, listening to music, eating together, having a party) due to physical distancing measures (NA 33) | 88.2% | 70.1% | 71.0% | 79.1% | 0.049 |
| Not being allowed to do something helpful for other residents due to physical distancing measures (NA 104, missing 3) | 57.9% | 58.1% | 67.2% | 68.6% | 0.682 |
| To have been requested to adopt physical distancing of at least 1 metre from others while participating to rehabilitation activities within the facility (NA 17, missing 2) | 48.7% | 64.1% | 66.7% | 53.9% | 0.150 |
| **Organisational changes** |  |  |  |  |  |
| The overall impact of the COVID-19 pandemic and related restrictive measures on the quality of your life (NA 9, missing 3) | 89.7% | 70.5% | 72.0% | 83.1% | 0.051 |
| To engage with remote consultations by phone or via digital platforms with GPs, treating psychiatrists or other therapists (NA 130, missing 2) | 65.0% | 57.7% | 61.9% | 53.8% | 0.796 |
| To be exclusively engaged in indoor rehabilitation activities due to interdiction of outdoor activities (NA 13, missing 3) | 53.8% | 64.4% | 50.5% | 67.1% | 0.120 |
| **control of Infection** |  |  |  |  |  |
| To adopt preventive measures within the facility, such as wear facial mask. use hand sanitiser gel, undergo triage procedures (NA 4, missing 2) | 58.5% | 58.7% | 55.4% | 65.4% | 0.607 |
| To regularly undergo nasopharyngeal swab (NA 12, missing 3) | 71.4% | 46.5% | 45.5% | 37.2% | 0.009 |
| To have the chance of being first in line for COVID-19 vaccination as a resident of a healthcare facility (NA 10, missing 2) | 24.3% | 20.5% | 14.7% | 13.0% | 0.382 |
